# Probable Tiger-to-Tiger Transmission of Avian Influenza H5N1

**DOI:** 10.3201/eid1105.050007

**Published:** 2005-05

**Authors:** Roongroje Thanawongnuwech, Alongkorn Amonsin, Rachod Tantilertcharoen, Sudarat Damrongwatanapokin, Apiradee Theamboonlers, Sunchai Payungporn, Kamonchart Nanthapornphiphat, Somchuan Ratanamungklanon, Eakchai Tunak, Thaweesak Songserm, Veravit Vivatthanavanich, Thawat Lekdumrongsak, Sawang Kesdangsakonwut, Schwann Tunhikorn, Yong Poovorawan

**Affiliations:** * Chulalongkorn University, Bangkok, Thailand;; † National Institute of Animal Health, Bangkok, Thailand;; ‡ Sriracha Tiger Zoo, Chonburi, Thailand;; § Eastern Veterinary Development Center, Chonburi, Thailand;; ¶ Kasetsart University, NakornPathom, Thailand;; # Chonburi Governor Office, Chonburi, Thailand;; ** National Park Wildlife and Plant Conservation Department, Bangkok, Thailand

**Keywords:** molecular epidemiology, Avian influenza H5N1, Tiger to tiger in transmission

## Abstract

During the second outbreak of avian influenza H5N1 in Thailand, probable horizontal transmission among tigers was demonstrated in the tiger zoo. Sequencing and phylogenetic analysis of those viruses showed no differences from the first isolate obtained in January 2004. This finding has implications for influenza virus epidemiology and pathogenicity in mammals.

In mid-January 2004, an epizootic outbreak of highly pathogenic avian influenza (HPAI H5N1 strain) was reported in poultry and various other birds in Thailand (1). Two tigers (*Panthera tigris*) and 2 leopards (*P. pardus*) in a zoo in Suphanburi, Thailand, died after experiencing high fever and respiratory distress; H5N1 infection was later confirmed as the cause of the illness (2). The animals had been fed raw chicken carcasses that were possibly contaminated with the HPAI H5N1 virus. A tiger zoo in Sriracha, Chonburi, Thailand, was affected by HPAI beginning on October 11, 2004.

## The Study

**Figure 1:**
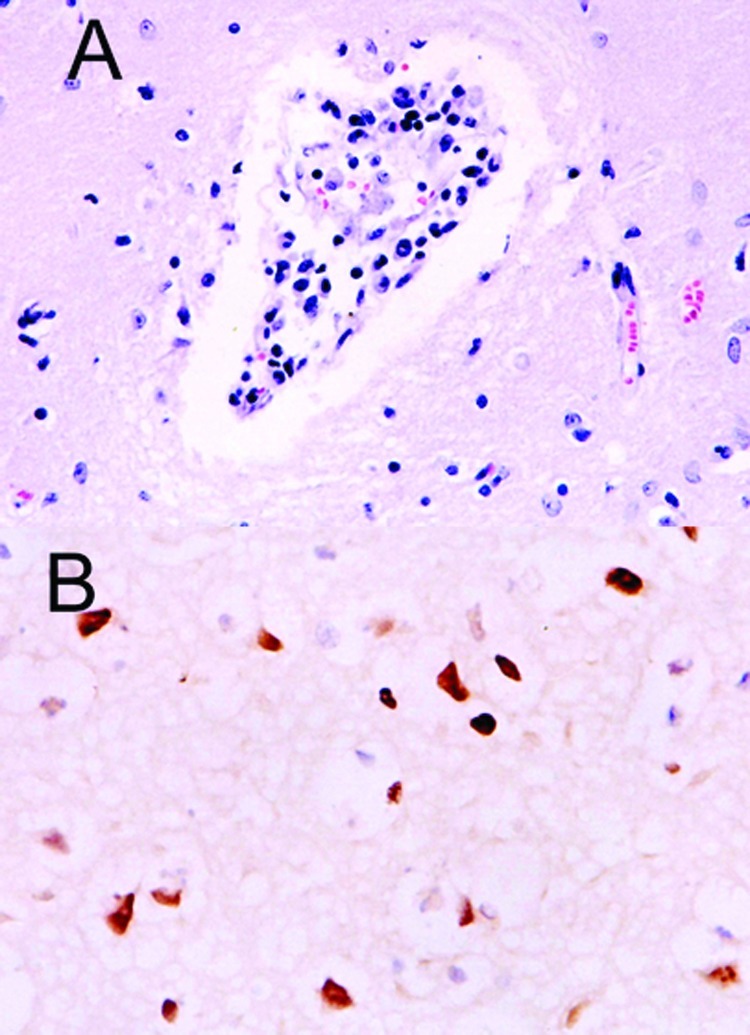
Histopathologic and immunohistochemical evidence of H5N1 virus in tiger: A) Mild multifocal nonsuppurative encephalitis; B) Influenza A virus antigen in nuclei and cytoplasm visible as brown staining.

The site of the HPAI outbreak is the biggest tiger zoo in Thailand, housing 441 tigers in 3 zones: breeder, nursery, and grower. The outbreak initially involved 16 tigers from 6 to 24 months of age in the grower zone. This zone was open for display; the other 2 zones were restricted areas. Laboratory findings in specimens from these animals included severe leukopenia and thrombocytopenia and increased levels of the liver enzymes alanine aminotransferase and aspartate aminotransferase (data not shown). Three days later, 5 tigers had died and 14 displayed varying degrees of clinical symptoms, including high fever and respiratory distress. All specimens submitted to the National Institute of Animal Health laboratory were positive for HPAI H5N1 by real-time polymerase chain reaction (PCR) and virus isolation and later confirmed by reverse transcriptase-PCR (3). On October 16, 2004, the tigers were fed cooked chicken carcasses or pork. Samples of raw chicken carcasses from the local suppliers were tested by using egg inoculation; 1 sample was positive for H5N1 virus. All animals that died had serosanguinous nasal discharge, and some had neurologic signs of infection. Necropsy was performed on 3 tigers on October 18. The lungs were severely congested and had been hemorrhaging. Serosanguinous exudate was seen throughout the tracheal and bronchiolar lumen, and pleural effusion was also seen. Microscopic findings showed moderate congestion of the brain with mild nonsuppurative meningoencephalitis (Figure 1A), severe diffuse lung hemorrhage and edema, and moderate multifocal necrotizing hepatitis. Immunohistochemical procedures were performed on all tissue by using mouse monoclonal antibody to the nucleoprotein of influenza A H5N1 (B.V. European Veterinary Laboratory, Woerden, the Netherlands). Strongly positive, brown staining was prominently displayed in the nuclei of the hepatocytes and in the cerebral neurons. Positive staining of both nuclei and cytoplasm was also apparent in the neurons (Figure 1B) and bronchiolar epithelium.

**Figure 2:**
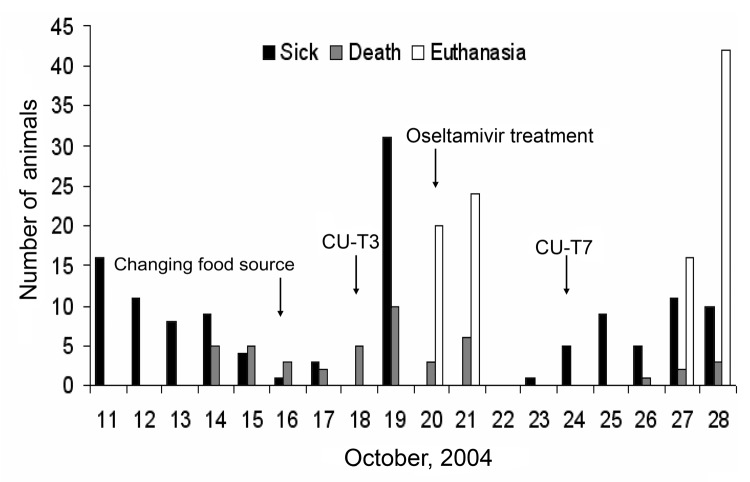
Numbers of sick, dead, or euthanized tigers during the outbreak. The animals were fed cooked chicken carcasses or pork after October 16, 2004. Isolates from the sick tigers, pre- and posttreated with oseltamivir, were A/Tiger/Thailand/CU-T3/04 (October 18) and A/Tiger/Thailand/CU-T7/04 (October 24).

Because the number of sick animals increased, the moribund animals were euthanized on October 20, 2004. Oseltamivir (Roche, Basel, Switzerland) (75mg/60 kg) was administered twice daily to the healthy tigers (4). On October 23, another sick tiger was found in the grower zone. The increasing numbers of sick tigers demonstrated the potential of tiger-to-tiger transmission (Figure 2). The duration of the disease was determined to be ≈3 days, since the infected tigers died 3 days after clinical signs were observed. Nasal swabs and blood samples were obtained from both infected and healthy tigers in the other 2 zones. Only 3 nasal swab samples from the infected zone were positive. Rectal swabs were then taken from the 3 sick animals; 1 was positive on egg inoculation. The remaining 42 tigers in the infected zone were euthanized to stop tiger-to-tiger transmission on October 28; a total of 147 tigers died or were euthanized. One week after the tigers in the grower zone were euthanized, 55 nasal swabs and serum samples (18.7%) obtained from the tigers in the other 2 zones were tested every other week for a 2-week period by using real time-PCR and egg inoculation. None of the samples was positive. Four weeks after the last tiger was infected, 58 serum samples were obtained from all humans that had been in contact with either the animals or their tissue or fluid, including veterinarians; these samples were tested twice by microneutralization for the H5N1 virus. Seroconversion (1:80, ≥1:640) occurred 6 weeks after the incident in only 2 (3.5%) persons who had shown no clinical signs of illness. To mitigate the risk for human infection and the potential for genetic reassortment, all persons involved in the incident were advised to take the commercially available human influenza vaccine (H3N2) for the 2003–2004 season.

Epidemiologic surveillance tests for the H5N1 virus were performed on the zoo animals and 11 wild avian species near the tiger zoo. Neither sickness nor death from the H5N1 virus had been observed in any of these animals before. Before reopening the zoo 3 weeks after the last infected tigers had died or been euthanized, water and soil samples or swabs from the tigers' cages were tested by using egg inoculation. None of the samples was positive.

Influenza A virus was isolated from the sick tigers' nasal swabs; A/Tiger/Thailand/CU-T3/04 (HA and NA gene; AY842935-6; PB2 and NS gene; AY907672-3) was initiated from those pretreated with oseltamivir, and A/Tiger/Thailand/CU-T7/04 (hemagglutinin [HA] and neuraminidase [NA] gene; AY866475-6, PB2; AY907671 and NS; AY907674) was isolated from those posttreated. Sequencing and phylogenetic analysis of the HA and NA genes of the H5N1 isolates in this outbreak showed that they were similar to each other as well as to those of the virus obtained from the earlier cases identified in 2004 (data not shown). The HA gene contained a glutamine at position 222 (226 in H3) in HA1 and 4 polybasic amino acid insertions at the cleavage site, which had also been found in other recent H5N1 isolates from chicken and tigers (1,2). However, no mutation of histidine to tyrosine was seen at position 274 of the NA molecule after oseltamivir treatment. In both isolates, a single amino acid substitution, glutamine to lysine, was observed at the position 627 in the PB2 protein responsible for H5N1 pathogenicity in mammals (5), and a 5-codon deletion was found in the NS gene, similar to the H5N1 viruses isolated in the same epidemic.

## Conclusions

The H5N1 virus is fatal to tigers, a conclusion also drawn in our previous report (2). The last case of the H5N1 virus infection was found in the tigers on October 28, 2004. The tigers had not been fed raw chicken carcasses in ≈12 days, and no other avian or mammal species kept in the zoo had been infected during this outbreak. Our results demonstrated that tigers kept in captivity are at risk for infection with and dying of the H5N1 virus; moreover, they could be infected by horizontal transmission since raw chicken carcasses represent the main food item for them. Alternative food for the tigers should be considered to reduce the risk for infection. Epidemiologic data obtained from this study demonstrated that all tigers that became ill after October 23, 2004, were probably infected by horizontal transmission since the animals had not been fed raw chicken carcasses since October 16. Administration of oseltamivir therapy could suppress and prolong the incubation period of the H5N1 virus infection, but it is unlikely.

To date, illness in tigers due to H5N1 infection is of the same severity as that in the H5N1 virus in cats (6). The serosanguinous nasal discharge seen in the sick tigers before death is likely due to severe thrombocytopenia. Results of laboratory findings, except liver enzyme levels, for the sick tigers were similar to the findings reported earlier in the pediatric cases (7). Positive staining for the NP protein of influenza A in the nuclei of the hepatocytes might indicate that a heavy virus load had passed through the digestive tract after the infected chicken carcasses were eaten, affecting the liver, particularly the hepatocytes, and possibly causing hepatic failure. Unlike results derived from experiments with cynomolgus monkeys (8), we were able to demonstrate H5N1 viral antigen in several organs of the infected tigers. The evidence of nonsuppurative encephalitis shown in the previous study (2) confirmed the involvement of H5N1 virus, as was apparent by using immunohistochemical procedures. H5N1 infection in tigers can induce neurologic signs and encephalitis similar to that observed in other mammals (9). Neurotropism of the H5N1 virus in mice as part of the pathogenesis subsequent to infection by human influenza virus isolates has been reported (10). Further studies will be required to elucidate the pathogenesis of the H5N1 virus in felines.

In vitro studies have demonstrated the potent antiviral activity of oseltamivir against all strains of influenza A tested, including the avian H5N1 virus recently implicated in human influenza cases in Hong Kong (11). In this study, the failure of treatment might have been attributable to various factors such as dosage, pharmacokinetics, or host metabolism, since no changes were seen in the neuraminidase. Application of antiviral therapy to those sick tigers has not been sufficiently researched and, hence, requires additional data.
