# Correction: Vol. 11, No. 5

**DOI:** 10.3201/eid1106.C31106

**Published:** 2005-06

**Keywords:** Correction, errata, erratum

In “Probable Tiger-to-Tiger Transmission of Avian Influenza H5N1,” by Roongroje Thanawongnuwech et al., an error occurred. The GenBank accession nos. for HA and NA gene initiated from influenza A virus (A/Tiger/ Thailand/CU-T3/04) are AY842935 and AY842936.

The corrected article appears online at http://wwwnc.cdc.gov/eid/article/11/5/05-0007_article.htm.

We regret any confusion these errors may have caused.

